# Tumor treating fields in glioblastoma: long-term treatment and high compliance as favorable prognostic factors

**DOI:** 10.3389/fonc.2024.1345190

**Published:** 2024-03-20

**Authors:** Junjie Wang, Quan Du, Jiarui Chen, Jianjian Liu, Zhaowen Gu, Xiaoyu Wang, Anke Zhang, Shiqi Gao, Anwen Shao, Jianmin Zhang, Yongjie Wang

**Affiliations:** ^1^ Department of Neurosurgery, Second Affiliated Hospital, School of Medicine, Zhejiang University, Hangzhou, Zhejiang, China; ^2^ Clinical Research Center for Neurological Diseases of Zhejiang Province, Hangzhou, China; ^3^ Department of Neurosurgery, Hangzhou First People’s Hospital, Hangzhou, China; ^4^ Brain Research Institute, Zhejiang University, Hangzhou, China; ^5^ Collaborative Innovation Center for Brain Science, Zhejiang University, Hangzhou, China

**Keywords:** glioblastoma, brain tumor, tumor treating fields, retrospective cohort, survival analysis

## Abstract

**Introduction:**

Tumor treating fields (TTFields) have earned substantial attention in recent years as a novel therapeutic approach with the potential to improve the prognosis of glioblastoma (GBM) patients. However, the impact of TTFields remains a subject of ongoing debate. This study aimed to offer real-world evidence on TTFields therapy for GBM, and to investigate the clinical determinants affecting its efficacy.

**Methods:**

We have reported a retrospective analysis of 81 newly diagnosed Chinese GBM patients who received TTFields/Stupp treatment in the Second Affiliated Hospital of Zhejiang University. Overall survival (OS) and progression-free survival (PFS) were analyzed using Kaplan–Meier method. Cox regression models with time-dependent covariates were utilized to address non-proportional hazards and to assess the influence of clinical variables on PFS and OS.

**Results:**

The median PFS and OS following TTFields/STUPP treatment was 12.6 months (95% CI 11.0-14.1) and 21.3 months (95% CI 10.0–32.6) respectively. Long-term TTFields treatment (>2 months) exhibits significant improvements in PFS and OS compared to the short-term treatment group (≤2 months). Time-dependent covariate COX analysis revealed that longer TTFields treatment was correlated with enhanced PFS and OS for up to 12 and 13 months, respectively. Higher compliance to TTFields (≥ 0.8) significantly reduced the death risk (HR=0.297, 95%CI 0.108-0.819). Complete surgical resection and MGMT promoter methylation were associated with significantly lower risk of progression (HR=0.337, 95% CI 0.176-0.643; HR=0.156, 95% CI 0.065-0.378) and death (HR=0.276, 95% CI 0.105-0.727; HR=0.249, 95% CI 0.087-0.710).

**Conclusion:**

The TTFields/Stupp treatment may prolong median OS and PFS in GBM patients, with long-term TTFields treatment, higher TTFields compliance, complete surgical resection, and MGMT promoter methylation significantly improving prognosis.

## 1 Introduction

Glioblastoma (GBM) stands out as a formidable and highly lethal disease, characterized by early recurrence and a gloomy prognosis (1–3). The multimodal approach to GBM treatment includes surgical intervention, radiotherapy, systemic chemotherapy and targeted therapy. Despite these combined therapeutic efforts, the outcomes remain stark, with most clinical investigations demonstrating a median overall survival (OS) of about 15 months (4, 5) and a 5-year survival rate of merely 5.8% (6, 7).

Tumor treating fields (TTFields) is an alternating electric field that deliver a low-intensity, mid-frequency electrical field of 200kHz, which impedes cell proliferation by disrupting the mitotic spindle, ultimately resulting in the disintegration of proliferating cells (8). TTFields has shown effectiveness in a range of studies, including *in vitro* cellular assays, *in vivo* animal models, and clinical trials involving GBM patients (9). Its promising therapeutic results have significantly changed first-line clinical management of GBM worldwide (10–12).

Recently, several clinical investigations, including the EF-14 randomized trial, have unequivocally exhibited that incorporating TTFields treatment with temozolomide (TMZ) chemotherapy yields a significant improvement in both PFS and OS of GBM patients (13–15). Although the results of these studies have led to the recommendation of TTFields treatment in GBM patients by Food and Drug Administration (FDA) and Chinese Glioma Cooperative Group (CGCG), there exist reports suggesting inconspicuous effectiveness of TTFields therapy (16). Owing to the skepticism induced by this variability in outcomes, some medical practitioners are adopting a cautious, observational stance (17, 18).

In this retrospective study, we evaluated a cohort of 81 newly diagnosed GBM patients treated with the TTFields/Stupp regimen, and investigated the impact of various clinical characteristics on both PFS and OS, especially focusing on treatment duration and patient compliance. This study aims to provide real-world evidence in guiding future applications of TTFields in GBM patients.

## 2 Methods

### 2.1 Patients and data collection

We recruited GBM patients who underwent surgery in several hospitals across Zhejiang Province from June, 2019 and December, 2022. After surgery, all these patients received the TTFields treatment and standard Stupp regimen at the Second Affiliated Hospital of Zhejiang University. Our study included patients meeting the following criteria: (1) age of 18 years or older, (2) histologically confirmed diagnosis of GBM, (3) newly diagnosed GBM, (4) TTFields treatment duration of at least 4 weeks. Informed consent was obtained from all patients. Approval was granted by the Ethics Committee of Second Affiliated Hospital, School of Medicine, Zhejiang University, Hangzhou, China, 313000 (No., 2023-1172).

After pathologically confirming the GBM diagnosis, all patients received the chemoradiotherapy regimen originally prescribed according to Stupp et al (4). Radiotherapy was given as daily dosage of 1.8-2.0 Gy for 30 fractions, with 2 days’ rest after every 5 days’ treatment. During radiotherapy, concurrent oral temozolomide was administered daily at a dose of 75mg/m² for 42 days. The adjuvant chemotherapy phase commenced 4 weeks later. Oral temozolomide was prescribed at a daily dosage of 150-200mg/m² for 5 consecutive days, with repetitions every 28 days, until completion of 12 cycles. TTFields treatment began either with or after radiotherapy and persisted until discontinuation due to tumor progression, financial limitations, patient refusal, or other factors.

Patients were routinely required to visit the outpatient clinic every 2 months for clinical evaluation and MRI scanning. If patients complained of any additional neurological symptoms, they would contact us for immediate examination. In cases when patients missed their appointment by more than 2 months, a telephone follow-up was initiated to assess their condition and remind them of the outpatient clinic appointment. The MRI scans were evaluated by two senior neuroradiologists to determine tumor progression. An independent third neuroradiologist (J.Z) would conduct an additional blinded evaluation if there was a disparity between the two senior neuroradiologists. The final results were selected according to the consensus between either two neurosurgeons.

All data collection was performed by the two neurosurgeons (X.W and A.Z). Baseline characteristics included age, gender, Karnofsky performance status score (KPS), extent of surgical resection, tumor location, TTFields treatment duration, patient compliance, progression-free survival (PFS), and overall survival (OS). Detailed review of medical records was performed for each participant in the trial. Comprehensive data on tumor pathology, treatments administered, and survival outcomes were compiled and analyzed. OS was defined as the interval from receiving surgical treatment to death from any cause, and PFS was defined as the time from receiving surgical treatment to confirmed disease progression based on imaging assessments. Gross total resection was determined by the surgical procedure and postoperative images. Specific pathological information on GBM was sourced from either our center’s pathology department or molecular laboratories. Compliance was assessed monthly based on the average daily usage percentage.

### 2.2 Statistical analysis

The Shapiro-Wilk test was used to test the normality. The median with the interquartile range from the first to the third quartile (Q1-Q3) were used for continuous variables with skewed distributions. Categorical variables were presented as number and percentage. Comparisons between groups were performed using the nonparametric Mann-Whitney U test for continuous variables, and Chi-square test or Fisher’s exact test for categorical parameters. Univariate Kaplan-Meier analyses employing both the log-rank test and Breslow test to compare difference in survival times. Clinical variables were assessed for proportional hazards assumption through time-dependent covariate analysis, with ln(time) as the defined time-dependent covariate. For clinical variables (X) that failed to meet the proportional hazards assumption, we constructed an interaction term [X*ln(time)] combining the variable (X) with the time-dependent covariate ln(time), and this term was then integrated into Cox regression model (19–22).

Continuous variables, including age, baseline KPS, usage time of TTFields, and average compliance of TTFields were turned into categorical variables: age ≥ 65, KPS >80, usage time of TTFields > 2 months, and average compliance of TTFields ≥ 0.8. The determination of cutoff values for the usage time and average compliance of TTFields was primarily based on findings from prior studies (23, 24). Variables with p values less than 0.10 in the univariate analysis were involved in the multivariate analysis utilizing Cox regression models with time-dependent covariate. All p-values were two-tailed, and a p-value < 0.05 was considered statistically significant. Analysis was performed using SPSS 26.0 (IBM Corporation, Armonk, NY, USA). Survival curves were generated using GraphPad Prism 9.0.0 (GraphPad Software, San Diego, CA, USA). Function graphs were generated using Origin, 2022 (OriginLab Corporation, Northampton, MA, USA).

## 3 Results

### 3.1 Study population

A total of 118 patients subjected to TTFields treatment at our institution between June, 2019 and December, 2022 was recruited. Following the exclusion of 13 patients with non-GBM pathologies, 17 patients diagnosed with secondary GBM, and 7 patients who became lost to follow-up, a resultant sample of 81 patients with newly diagnosed GBM and subjecting to TTFields therapy were included in this investigation. None of the patients had a history of other major comorbidities. The median follow-up of this study was 18.9 months (95% CI 14.3-23.5). The detailed demographic and clinical data were listed in **Table 1**.

**Table 1:** Patient characteristics.

		81 GBM patients receiving TTFields treatment			
		Total (n = 81)	TTFields time ≤ 2 m (n = 22)	TTFields time> 2 m (n = 59)	*p*-Value
**Gender**	Female	40 (49.4)	9 (40.9)	31 (52.5)	0.352
**Age (y)**	Median (Q1-Q3)	55 (42-64)	59.5 (46.75-68.25)	55 (40-64)	0.226
	Age ≥ 65	19 (23.5)	7 (31.8)	12 (20.3)	0.278
**Educational background**	High school degree & above	45 (55.6)	9 (40.9)	36 (61.0)	0.105
**Baseline KPS**	Median (Q1-Q3)	90 (90-90)	90 (70-90)	90 (90-90)	0.002
	KPS > 80	65 (80.2)	13 (59.1)	52 (88.1)	0.009
**Tumor location**	Frontal lobe	18 (22.2)	8 (36.4)	10 (16.9)	0.089
	Superficial hemisphere	48 (59.3)	9 (40.9)	39 (66.1)	
	Midline/deep structure/infratentorial	15 (18.5)	5 (22.7)	10 (16.9)	
**Extent of surgical resection**	Gross total resection	36 (44.4)	9 (40.9)	27 (45.8)	0.696
**IDH mutant status**	Wild type	75 (92.5)	21 (95.5)	54 (91.5)	1
	Mutated	5 (6.2)	1 (4.5)	4 (6.8)	
	Invalid	1 (1.2)	0	1 (1.7)	
**MGMT promoter methylation**	Unmethylated	33 (40.7)	12 (54.5)	21 (35.6)	0.304
	Methylated	24 (29.6)	5 (22.7)	19 (32.2)	
	Invalid	24 (29.6)	5 (22.7)	19 (32.2)	
**TERT promoter mutation**	Wild type	22 (27.2)	7 (31.8)	15 (25.4)	0.845
	Mutated	32 (39.5)	8 (36.4)	24 (40.7)	
	Invalid	27 (33.3)	7 (31.8)	20 (33.9)	
**Average compliance of TTFields**	Median (Q1-Q3)	0.9 (0.8-0.9)	0.8 (0.79-0.88)	0.9 (0.84-0.9)	0.001
	Average compliance ≥ 0.80	71 (87.7)	16 (72.7)	55 (93.2)	0.035

Among 81 patients enrolled, 49.4% were female. The median age was 55 years (Q1-Q3 42-64), with 19% of individuals being aged 65 or older. The median Karnofsky performance status score (KPS) of the patients was 90 (Q1-Q3 90-90), and 80.2% of them exhibited a baseline KPS exceeding 80. Tumor localization was classified into frontal (22.2%), superficial excluding the frontal lobe (59.3%), and midline/deep structure/infratentorial (18.5%) regions. Additionally, gross total tumor resection (accessed by intraoperative assessment and postoperative imaging) was accomplished in 44.4% of patients. IDH mutant status, MGMT methylation, and TERT promoter were evaluated based on patients’ pathology reports. After surgery, all patients underwent the recommended TTFields therapy alongside standard temozolomide chemotherapy and radiotherapy (Stupp regimen).

The analysis of TTFields application data revealed that among 81 patients, 59 (72.8%) used TTFields for more than 2 months, with median duration of 7 months (Q1-Q3 2-11). Moreover, 71 patients (87.8%) attained an average compliance rate exceeding 0.8, with a median compliance of 0.9 (Q1-Q3 0.8-0.9). Univariate analysis revealed significant difference in baseline KPS and average TTFields compliance between long-term TTFields group (>2 months) and short-term TTFields group (≤2 months). The KPS scores and the percentage of patients with KPS >80 were considerably greater in the long-term TTFields group compared to short-term group (p=0.002 and p=0.009, respectively). Additionally, the long-term TTFields group (>2 months) exhibited higher treatment compliance and a larger fraction of patients with high compliance (≥0.80) compared to their short-term (≤2 months) counterparts (p=0.001 and p=0.035). Moreover, no significant difference was found between the groups in terms of gender, age, educational background, other tumor characteristics, and surgical methods.

### 3.2 Univariate Kaplan-Meier analyses

**Figure 1:**
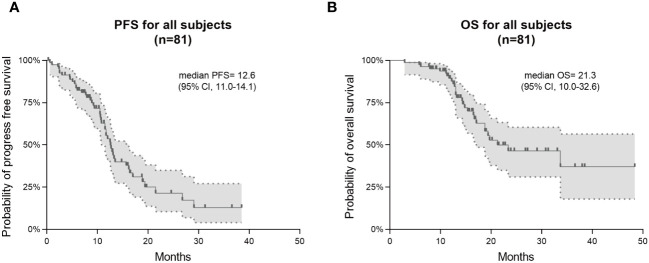
Kaplan–Meier survival curves in PFS and OS for all 81 GBM patients. Progression-free survival (PFS) is shown in **(A)** and overall survival (OS) in **(B)**.

This study found that the median PFS and median OS for patients receiving TTFields along with the Stupp protocol were 12.6 months (95% CI 11.0-14.1) and 21.3 months (95% CI 10.0-32.6), respectively (**Figure 1**). Kaplan-Meier analyses of PFS and OS were performed across multiple clinical parameters, as detailed in **Table 2**. Usage time of TTFields, average compliance of TTFields, extent of surgical resection, and MGMT promoter methylation were significantly associated with patients’ survival time.

**Table 2:** The Kaplan-Meier analyses for PFS and OS in GBM patients.

		PFS			OS		
		Median PFS (95% CI)	Log-rank test *p*-value	Breslow test *p*-value	Median OS (95% CI)	Log-rank test *p*-value	Breslow test *p*-value
**Gender**	Male	12.7 (11.1-14.2)	0.953	0.573	19.4 (14.6-24.3)	0.79	0.928
	Female	12.0 (9.5-14.5)			23.4 (15.4-NA)		
**Age (y)**	< 65	12.9 (9.2-16.5)	0.287	0.315	33.7 (15.6-51.8)	0.546	0.933
	≥ 65	11.5 (9.9-13.1)			18.8 (13.3-24.3)		
**Baseline KPS**	KPS ≤ 80	10.6 (3.0-18.1)	0.341	0.052	19.7 (15.0-24.5)	0.148	0.064
	KPS >80	12.6 (10.9-14.2)			33.7 (11.6-55.8)		
**Tumor location**	Frontal lobe	12.0 (10.8-13.2)	0.752	0.923	NR	0.688	0.984
	Superficial hemisphere	13.1 (10.7-15.5)			19.4 (14.8-24.0)		
	Midline/deep structure/infratentorial	18.9 (9.9-27.9)			19.7 (11.0-28.5)		
**Extent of surgical resection**	Partial resection/Biopsy	10.6 (10.2-10.9)	0.027	0.002	18.8 (15.6-22.0)	0.016	0.031
	Gross total resection	16.2 (11.7-20.7)			NR		
**IDH mutant status**	Wild type	12.5 (11.2-13.7)	0.398	0.544	19.7 (16.7-NA)	0.325	0.305
	Mutated	29.1 (0.0-67.0)			NR		
	Invalid	NA			NA		
**MGMT promoter methylation**	Unmethylated	9.4 (6.9-11.9)	<0.001	<0.001	18.8 (12.8-24.8)	0.037	0.05
	Methylated	26.8 (11.5-42.2)			NR		
	Invalid	13.3 (12.0-14.5)			33.7 (0.0-NA)		
**TERT promoter mutation**	Wild type	10.7 (8.0-13.4)	0.223	0.107	NR	0.44	0.282
	Mutated	12.5 (10.8-14.1)			21.3 (13.1-29.6)		
	Invalid	13.1 (7.0-19.2)			19.7 (14.7-24.7)		
**Usage time of TTFields**	≤ 2 months	11.5 (5.3-17.7)	0.118	0.009	21.3 (11.3-31.3)	0.389	0.049
	> 2 months	13.1 (11.9-14.3)			23.4 (12.0-34.8)		
**Average compliance of TTFields**	< 0.8	11.5 (10.3-12.7)	0.831	0.705	16.5 (10.4-22.7)	0.001	0.002
	≥ 0.8	12.7 (11.4-14.0)			33.7 (14.5-52.9)		

NR, Not Reached; NA, Not Applicable.

Patients from the long-term TTFields group (>2 months) exhibited significantly longer PFS and OS compared to those from the short-term group, with Breslow p-values of 0.009 for PFS and 0.049 for OS. The median PFS was 13.1 months (95% CI 11.9-14.3) for the long-term group and 11.5 months (95% CI 5.3-17.7) for the short-term group. Median OS was 23.4 months (95% CI 12.0-34.8) for the long-term group and 21.3 months (95% CI 11.3-31.3) for the short-term group. In terms of average compliance with TTFields, there was a remarkable extension of OS in the high-compliance group (≥0.80) as compared to the low-compliance group (<0.80). The median OS was 33.7 months (95% CI 14.5-52.9) and 16.5 months (95% CI 10.4-22.7) in high- and low-compliance groups respectively (Log-rank p=0.001, Breslow p=0.002). However, PFS did not differ significantly between these two groups.

The extent of surgical resection was significantly correlated with both PFS (Log-rank p=0.027, Breslow p=0.002) and OS (Log-rank p=0.016, Breslow p=0.031). Patients with incomplete resections had a median PFS of 10.6 months (95% CI 10.2-10.9), while patients with complete resections had a median PFS of 16.2 months (95% CI 11.7-20.7). In terms of OS, patients who received incomplete resection had a median OS of 18.8 months (95% CI 15.5-22). Compared to this, patients who underwent complete resection had a longer OS, and did not reach the median overall survival during the follow-up period.

The different methylation status of MGMT promoter showed significant statistical difference in both PFS (Log-rank p<0.001, Breslow p <0.001) and OS (Log-rank p=0.037, Breslow p=0.05). The PFS and OS were significantly longer in the MGMT promoter-methylated group compared to the non-methylated group. During the follow-up period, the MGMT promoter-methylated group’s median OS was not attained, and the non-methylated group demonstrated a median OS of 18.8 months (95% CI 12.8-24.8). The median PFS for the two groups were 26.8 months (95% CI 11.5-42.2) and 9.4 months (95% CI 6.9-11.9), respectively.

Besides, no significant differences in PFS or OS were observed in relation to mutations in other molecular markers, including IDH and the TERT promoter. And other factors such as gender, age, baseline KPS, and tumor location were not significantly associated with survival times in the Kaplan-Meier analysis.

### 3.3 Proportional hazards assumption

Before incorporating any clinical variables into the multivariate COX model, we must ensure that each variable meets the proportional hazards assumption (**Table 3**). The analysis showed that the usage time of TTFields was significantly associated with the time-dependent covariate for both PFS and OS (p-values of 0.018 and 0.032, respectively), thereby violating the proportional hazards assumption. Additionally, other clinical variables, including Baseline KPS, extent of surgical resection, MGMT promoter methylation, and average compliance of TTFields, met the proportional hazards assumption as they did not show a significant association with the time-dependent covariate.

**Table 3:** Using time-dependent covariate to test whether each clinical factor met the proportional hazards assumption.

		PFS		OS	
		Time-dependent covariate			
		HR (95% CI)	*p*-value	HR (95% CI)	*p*-value
**Baseline KPS**	KPS ≤ 80	1.61 (0.72-3.56)	0.244	6.77 (0.758-60.48)	0.087
	KPS >80				
**Extent of surgical resection**	Partial resection/Biopsy	2.735 (0.87-8.65)	0.087	1.99 (0.24-16.37)	0.523
	Gross total resection				
**MGMT promoter methylation**	Unmethylated	1.49 (0.81-2.76)	0.203	1.36 (0.63-2.96)	0.437
	Methylated				
	Invalid				
**Usage time of TTFields**	≤ 2 months	3.35 (1.23-9.13)	0.018	46.77 (1.39-1573.73)	0.032
	> 2 months				
**Average compliance of TTFields**	< 0.8	2.63 (0.89-7.77)	0.079	2.43 (0.27-21.79)	0.429
	≥ 0.8				

### 3.4 Multivariate analysis

Clinical variables showing a Log-rank or Breslow p-value below 0.1 in univariate Kaplan-Meier analysis were selected for multivariate analysis. The usage time of TTFields was integrated with time-dependent covariates to develop multivariate Cox models to analyze these included clinical variables (**Tables 4**, **5**).

**Table 4:** Multivariate COX model with Time-dependent covariate for PFS.

	B	HR	Multivariate Analysis		Adjusted *p*-Value
			95% CI		
			Lower	Upper	
Usage time of TTFields					
≤ 2 months		1			
> 2 months	-3.243	0.039	0.004	0.344	0.003
**Time-dependent covariate**	1.282	3.604	1.323	9.820	0.012
Baseline KPS					
≤ 80		1			
> 80	0.819	2.269	0.993	5.182	0.052
Extent of surgical resection					
Partial resection/Biopsy		1			
Gross total resection	-1.089	0.337	0.176	0.643	0.001
MGMT promoter methylation					
Unmethylated		1			
Methylated	-1.855	0.156	0.065	0.378	<0.001
Invalid	-1.054	0.349	0.165	0.738	0.006

**Table 5:** Multivariate COX model with Time-dependent covariate for OS.

	B	HR	Multivariate Analysis		Adjusted *p*-Value
			95% CI		
			Lower	Upper	
Usage time of TTFields					
≤ 2 months		1			
> 2 months	-10.549	0.000	0.000	0.313	0.028
**Time-dependent covariate**	4.095	60.043	1.596	2258.515	0.027
Baseline KPS					
≤ 80		1			
> 80	0.373	1.452	0.542	3.891	0.459
Extent of surgical resection					
Partial resection/Biopsy		1			
Gross total resection	-1.289	0.276	0.105	0.727	0.009
MGMT promoter methylation					
Unmethylated		1			
Methylated	-1.391	0.249	0.087	0.710	0.009
Invalid	-0.853	0.426	0.166	1.096	0.077
Average compliance of TTFields					
< 0.8		1			
≥ 0.8	-1.213	0.297	0.108	0.819	0.019

**Figure 2:**
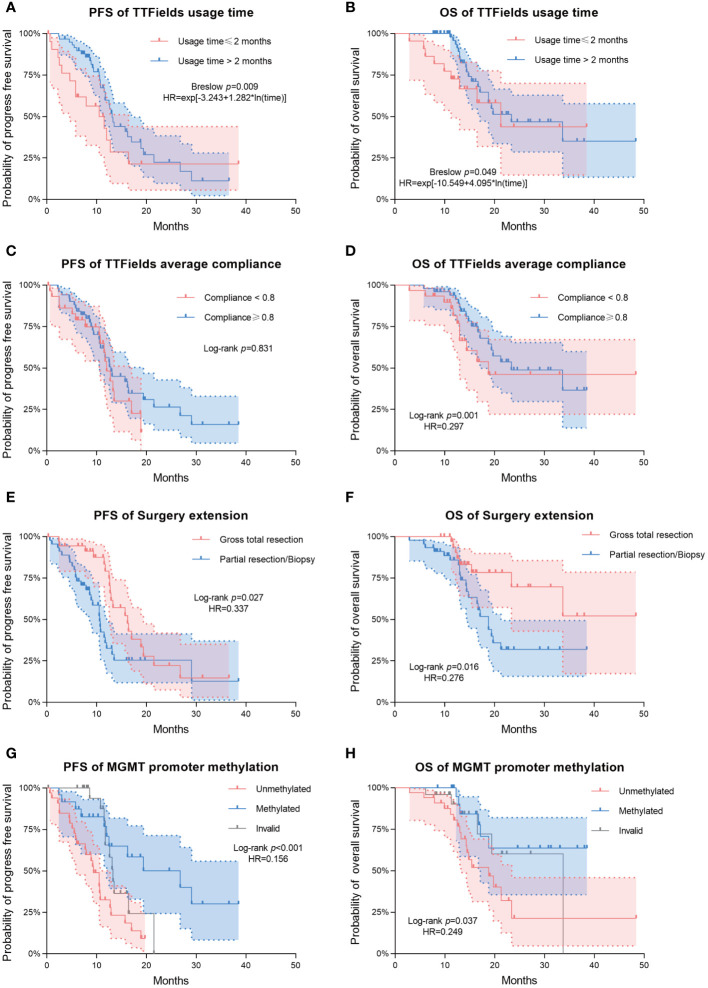
Kaplan–Meier survival analysis for comparing the prognostic impact of various clinical variables. Progression-free survival (PFS) and overall survival (OS) are shown in the first and second columns of the figure, respectively. **(A, B)** Differences in PFS/OS among TTFields usage time groups. **(C, D)** Differences in PFS/OS among TTFields average compliance groups. **(E, F)** Differences in PFS/OS among surgery extension groups. **(G, H)** Differences in PFS/OS among MGMT promoter methylation groups. Dashed lines represent estimated 95% confidence intervals of the hazard ratios (HRs).

**Figure 3:**
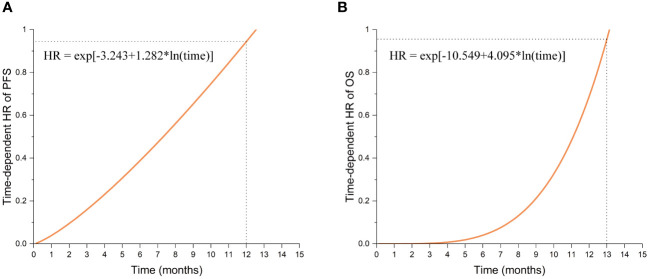
The function graphs of time-dependent HR values for Long-term TTFields treatment (>2 months). **(A)** The time-dependent HR values in progression-free survival (PFS). **(B)** The time-dependent HR values in overall survival (OS).

Multivariate analysis indicated significant effects of TTFields usage duration on both PFS and OS (p= 0.003 and 0.028, respectively). Given the violation of the proportional hazards assumption, the hazard ratios (HRs) for TTFields usage were time-variant, with the long-term TTFields usage group (>2 months) exhibiting HRs below 1 before 12 and 13 months of follow-up (**Figures 2A, B**, **3**). These findings imply that, over a follow-up period of approximately one-year, long-term TTFields therapy (>2 months) substantially decreases the risk of tumor progression and mortality relative to short-term therapy (≤ 2 months). Additionally, the high-compliance group (≥ 0.80) exhibited a markedly lower mortality risk than the low-compliance group (<0.80) (OS HR=0.297, 95% CI 0.108-0.819, p=0.019) (**Figure 2D**).

The patients who underwent complete tumor resection had significantly lower risks of tumor progression and death (PFS HR=0.337, 95% CI 0.176-0.643, p=0.001; OS HR=0.276, 95% CI 0.105-0.727, p=0.009) (**Figures 2E, F**). Likewise, the MGMT promoter-methylated cohort exhibited significantly lower risks of progression and mortality than their unmethylated counterparts (PFS HR=0.156, 95% CI 0.065-0.378, p<0.001; OS HR=0.249, 95% CI 0.087-0.710, p=0.009) (**Figures 2G, H**). Additionally, multivariate analysis showed that neither high (>80) nor low (≤ 80) baseline KPS significantly affected patient survival outcomes (PFS p-value = 0.052; OS p-value = 0.459).

## 4 Discussion

GBM is one of the deadliest and most challenging diseases, with the majority of reports demonstrating a median PFS of 6.2-7.5 months and a median OS of only 14.6-16.7 months (13). Prior clinical studies have highlighted the potential of TTFields therapy, an innovative and promising treatment, to significantly improve both PFS and OS for GBM patients when combined with Stupp regimen (13, 14). Nevertheless, TTFields has not gained essential global recognition as the established standard of care for primary GBM treatment, and it has not yet found its place within the guideline of the European Association of Neuro-Oncology treatment (15). In this retrospective study, we analyzed a total of 81 newly diagnosed GBM patients who underwent TTFields/Stupp regimen, and investigated the impact of various clinical characteristics on both OS and PFS. We hope that the results of this study will provide valuable evidence to inform future treatment strategies for GBM.

The therapeutic efficacy of TTFields/Stupp regimen remains a prominent focus. Taking a comparative lens, the EF-14 trial delineated a median PFS of 6.7 months (95% CI 6.1-8.1) and OS of 20.9 months (95% CI 19.3-22.7) for patients treated with TTFields/Stupp regimen (13). Another study from China posited a median PFS of 16 months (95% CI 9.6-24.6) and OS of 21.8 months (95% CI 17.4-NA) (14). In our study, the median PFS was 12.6 months (95% CI 11.0-14.1), positioning it between the results of the two aforementioned studies. The median overall survival (OS) in our cohort was 21.3 months (95% CI 10.0-32.6), aligning closely with the outcomes of the previously mentioned studies. These comparisons demonstrated the consistent effects of TTFields/Stupp regimen across different cohorts. The observed median OS in our cohort was 21.3 months. Although our study lasks a control group for Stupp-only treatment, we could compare our data to the previously reports as reference, which showed median OS of 16 and 15 months with Stupp treatment alone (13, 14). This result suggests that integrating TTFields with the Stupp regimen could markedly enhance the survival prognosis for GBM patients.

### 4.1 Impact of TTFields therapy duration on survival outcomes

The Kaplan-Meier survival analysis revealed that patients in the long-term TTFields usage group (>2 months) exhibited improved prognoses compared to those in the short-term usage group (≤ 2 months) (**Figures 2A, B**, **Table 2**). The median PFS in the long-term group (>2 months) was 13.1 months (95% CI 11.9-14.3) versus 11.5 months (95% CI 5.3-17.7) in the short-term group (≤ 2 months) (Breslow p = 0.009). The median OS reached 23.4 months (95% CI 12.0-34.8) in the long-term group (>2 months), in contrast to 21.3 months (95% CI 11.3-31.3) in the short-term group (≤ 2 months) (Breslow p = 0.049). The Breslow test alone reached statistical significance, unlike the Log-rank test, indicating more pronounced early follow-up prognostic differences between these groups (25). Analysis revealed that the usage time of TTFields treatment did not satisfy the proportional hazards assumption (**Table 3**). Time-dependent multivariate Cox analysis revealed that long-term TTFields usage (>2 months) was significantly associated with both progression (p=0.003) and mortality risks (p=0.028) (**Tables 4**, **5**), and that the HRs increased gradually, approaching 1 at 12 and 13 months of follow-up (**Figure 3**). The usage time of TTFields treatment has a higher HR during early follow-up, and this finding aligns with the results of the univariate K-M analysis, where only the Breslow test reached statistical significance. We speculate that this might be due to the poor prognostic characteristics of GBM. As postoperative recurring tumors gradually proliferate, the heterogeneity of the tumor changes, and the therapeutic efficacy of TTFields also gradually diminishes (26).

In summary, our study is the first report demonstrating that GBM patients undergoing long-term TTFields treatment (>2 months) exhibit a statistically significant improved PFS and OS compared to the short-term treatment group (≤2 months). Although the protective benefits of long-term TTFields treatment appear to diminish over time, they still convey a reduced risk of progression and death during the follow-up exceeding 12 months. Glas and colleagues conducted a radiological study on GBM patients who used TTFields for more than 2 months, finding that TTFields suppressed tumor progression in a dose-dependent manner (23). This aligns with our findings, endorsing the advantages of prolonged and higher-dose TTFields therapy in GBM patients. Interestingly, in our cohort, patients treated with the short-term TTFields (≤2 months) had a median PFS and OS of 11.5 months and 21.3 months, respectively. This outcome still surpasses the prognosis of Stupp-only treatment. As a matter of fact, the majority of patients (95.5%) in the short-term TTFields treatment group (≤ 2 months) completed the 2-month regimen, indicating a 2-month duration of TTFields treatment may confer significant therapeutic benefits. Further research with control groups and larger cohorts is necessary to confirm this hypothesis.

### 4.2 Impact of TTFields therapy compliance on survival outcomes

The compliance with TTFields therapy has been shown to be a critical determinant influencing patients’ outcomes (27, 28). The PRiDe study suggested a median compliance rate of 75% for TTFields (using the device for ≥18 hours per day) (24). According to the actr-27 study, an average monthly compliance of at least 50% was necessary to meaningfully prolong PFS and OS, with higher compliance correlating with improved prognoses (29). The EF-14 phase 3 trial reported a compliance rate of 75% and a median PFS of 6.7 months (13), whereas our cohort achieved a higher compliance rate of 90% and a prolonged median PFS of 12.6 months. However, a Chinese study with a median compliance rate of 85% and median PFS of 16 months (14), along with another study of only 16 GBM patients showing a compliance rate of 83% and median PFS of 20 months (30), highlights the variability in PFS outcomes across different studies. Our data, corroborated by previous studies (31), indicate that high TTFields treatment compliance may prolong PFS. Yet, given the inter-center variations in median PFS, larger multicenter studies are required. With respect to OS, our results revealed a median OS of 21.3 months, consistent with the phase 3 EF-14 trial’s median OS of 20.9 months and a Chinese center’s 21.8 months (13, 14). These findings support the association of high compliance to TTFields with increased OS.

Furthermore, we examined the prognostic disparities between patient groups with compliance rates below 0.8 and those 0.8 or above. PFS did not significantly differ between the groups (Log-rank p=0.831). However, a significant disparity was observed in OS (Log-rank p=0.001). Patients with ≥0.8 compliance had a median OS of 33.7 months (95% CI 14.5-52.9), in stark contrast to a median OS of 16.5 months in patients with <0.8 compliance (95% CI 10.4-22.7) (**Table 2**, **Figures 2C, D**). The multivariate COX model for OS (**Table 5**) suggested that higher compliance to TTFields (≥ 0.8) significantly reduces the risk of death (HR=0.297, 95%CI 0.108-0.819). In summary, our study confirms that long-term TTFields treatment and high compliance to TTFields therapy significantly improve the prognosis of GBM patients. Therefore, clinicians should emphasize the importance of maintaining TTFields treatment protocols with patients.

### 4.3 Other clinical variables affecting survival outcomes

Our study found no significant prognostic correlation with clinical variables such as gender, age, baseline KPS, or tumor location. However, the extent of surgical resection and MGMT promoter methylation were significantly associated with prognosis in both univariate and multivariate analysis.

Regarding surgical resection, patients undergoing gross total resection demonstrated significantly improved PFS and OS compared to those with partial resection/biopsy (**Figures 2E, F**), with reduced risk of progression (HR=0.337, 95% CI 0.176-0.643) and death (HR=0.276, 95% CI 0.105-0.727). This aligns with prior research indicating that complete tumor resection can substantially lessen tumor burden, thus benefiting GBM patient outcomes (24).

In the context of molecular biomarkers for tumors, our findings indicated a significant increase in both PFS and OS associated with MGMT promoter methylation (**Figures 2G, H**). Specifically, MGMT promoter methylation resulted in a marked decrease of recurrence risk (HR=0.156, 95% CI 0.065-0.378) and mortality risk (HR=0.249, 95% CI 0.087-0.710), which corroborates existing literature on TTFields therapy (32, 33). Patients with MGMT-methylated tumors had superior prognoses, potentially attributable to increased temozolomide (TMZ) sensitivity in these tumors (34). Notably, recent research on human GBM cell lines indicated a synergistic effect of combining TTFields with chemotherapy agents such as TMZ on MGMT promoter methylated cells (35). Moreover, our study found no significant prognostic impact from other tumor biomarkers, such as IDH and TERT promoter mutations. Although TERT promoter mutations typically correlate with an unfavorable prognosis (36, 37), our study revealed that patients harboring these mutations attained a median OS of 21.3 months after TTFields therapy, which was comparable with that of the wild-type group (Log-rank p=0.44). This indicates that TTFields therapy could potentially neutralize the adverse prognostic impact of TERT promoter mutations.

### 4.4 Study limitations

The current study included only the patients treated with TTFields in addition to Stupp strategy, and lacked a control group receiving only standard stupp treatment. Some patients were not subjected to a comprehensive molecular analysis, leading to incomplete pathological data.

Future studies should enlarge the sample size and include comparative analyses with control group to substantiate the conclusions. Additionally, future research should use multi-omics analyses of pathology (exome sequencing, methylation, RNA sequencing, etc.) to further classify tumors and identify patient groups most likely to benefit from TTFields treatment.

## 5 Conclusion

In conclusion, this single-center retrospective study from China included 81 newly diagnosed GBM patients treated with TTFields. The findings indicated that combining TTFields with Stupp treatment potentially extends the median OS (21.3 months) and median PFS (12.6 months) for GBM patients, with higher compliance (≥ 0.8) and prolonged TTFields usage (>2 months) correspondingly improving prognosis. Furthermore, GBM patients with complete surgical resection and MGMT promoter methylation demonstrated an enhanced prognosis.

## Data availability statement

The raw data supporting the conclusions of this article will be made available by the authors, without undue reservation.

## Ethics statement

The studies involving humans were approved by the Ethics Committee of Second Affiliated Hospital, School of Medicine, Zhejiang University, China, 313000. The studies were conducted in accordance with the local legislation and institutional requirements. The participants provided their written informed consent to participate in this study.

## Author contributions

JW: Writing – original draft, Formal analysis. QD: Writing – original draft, Methodology. JC: Writing – review & editing, Formal analysis. JL: Writing – review & editing, Data curation. ZG: Writing – review & editing, Data curation. XW: Writing – review & editing, Investigation. AZ: Writing – review & editing, Investigation. SG: Writing – review & editing. AS: Writing – review & editing. JZ: Writing – review & editing, Supervision, Conceptualization. YW: Writing – review & editing, Methodology, Funding acquisition, Conceptualization.
